# Immune Privilege and Eye-Derived T-Regulatory Cells

**DOI:** 10.1155/2018/1679197

**Published:** 2018-05-20

**Authors:** Hiroshi Keino, Shintaro Horie, Sunao Sugita

**Affiliations:** ^1^Department of Ophthalmology, Kyorin University School of Medicine, Tokyo, Japan; ^2^Department of Ophthalmology and Visual Science, Tokyo Medical and Dental University Graduate School of Medical and Dental Sciences, Tokyo, Japan; ^3^Laboratory for Retinal Regeneration, Center for Developmental Biology, Riken, Kobe, Japan

## Abstract

Certain cellular components of the eye, such as neural retina, are unable to regenerate and replicate after destructive inflammation. Ocular immune privilege provides the eye with immune protection against intraocular inflammation in order to minimize the risk to vision integrity. The eye and immune system use strategies to maintain the ocular immune privilege by regulating the innate and adaptive immune response, which includes immunological ignorance, peripheral tolerance to eye-derived antigens, and intraocular immunosuppressive microenvironment. In this review, we summarize current knowledge regarding the molecular mechanism responsible for the development and maintenance of ocular immune privilege via regulatory T cells (Tregs), which are generated by the anterior chamber-associated immune deviation (ACAID), and ocular resident cells including corneal endothelial (CE) cells, ocular pigment epithelial (PE) cells, and aqueous humor. Furthermore, we examined the therapeutic potential of Tregs generated by RPE cells that express transforming growth factor beta (TGF-*β*), cytotoxic T lymphocyte-associated antigen-2 alpha (CTLA-2*α*), and retinoic acid for autoimmune uveoretinitis and evaluated a new strategy using human RPE-induced Tregs for clinical application in inflammatory ocular disease. We believe that a better understanding of the ocular immune privilege associated with Tregs might offer a new approach with regard to therapeutic interventions for ocular autoimmunity.

## 1. Introduction

The microenvironment in the eye is both immunosuppressive and anti-inflammatory in nature. This immunosuppressive property by ocular resident cells/tissues is referred to as immune privilege. This phenomenon helps prevent extensive damage caused by infiltrating inflammatory cells that would otherwise lead to blindness. The eye expresses an extensive array of mechanisms through which innate and adaptive immune cells can be regulated, thereby avoiding blindness as a consequence of intraocular inflammation [1–3]. The immunosuppressive mechanisms that have been revealed to date include a microenvironment in the eye, for example, ocular fluids, blood-retina barriers, and ocular resident parenchymal cells. Ocular fluids, which include aqueous humor and vitreous fluids, have anti-inflammatory properties [4–6]. Some ocular resident cells create a blood-retina barrier to limit the ingress of blood cells, while ocular parenchymal cells express the CD95 ligand (CD95L/Fas ligand) that triggers apoptosis of inflammatory cells [7]. In these ocular immune privilege cells, retinal pigment epithelial (RPE) cells contribute to the immune privilege property of the eye. RPE cells form tight junctions and create the blood-retina barriers. Moreover, RPE cells constitutively express immunosuppressive molecules and secrete soluble immunomodulatory factors that are capable of mediating immunogenic inflammation [8, 9]. These mechanisms make it possible for the eye to regulate the intraocular innate and adaptive inflammatory response and accept transplanted tissue grafts for extended periods. In contrast, conventional body sites summarily reject such grafts. This review focuses on the development and maintenance of the immunosuppressive intraocular microenvironment formed via the generation of regulatory T cells (Tregs) by anterior chamber-associated immune deviation (ACAID), and ocular resident cells, which include corneal endothelial (CE) cells, ocular pigment epithelial (PE) cells, and aqueous humor. This review also evaluated the therapeutic potential of Tregs as powerful immunosuppressive cells that can be used for active noninfectious uveitis and corneal allograft transplantation.

## 2. Generation of Tregs in Eye-Derived Tolerance

To achieve immune privilege, the eye uses several different strategies to prevent and regulate sight-destroying inflammation in the eye [1, 10]. One of the strategies is the induction of the peripheral tolerance of eye-derived antigens referred to as ACAID [1, 11]. Antigenic materials in the anterior chamber generate a systemic immune response that retains primed, clonally expanded cytotoxic T-cell precursors and B cells secreting large concentrations of IgG_1_, which is a non-complement-fixing antibody. On the other hand, ACAID inhibits CD4^+^ Th1 and Th2 cells and B cells secreting complement-fixing antibodies [1, 2, 12–16]. The spleens of mice that receive antigen in the anterior chamber acquire three types of antigen-specific Tregs that mediate ACAID [17–19]. One of these populations consists of CD4^+^ T cells, which are known as the “afferent regulators,” as these CD4^+^ T cells are able to suppress the initial activation and differentiation of naïve T cells into Th1 effector cells. The second population consists of CD8^+^ T cells, which are known as “efferent regulators,” as these CD8^+^ T cells inhibit the expression of Th1 immune responses such as delayed hypersensitivity. The third population consists of CD8^+^ T cells that inhibit B cells from switching to the IgG isotype that fixes the complement. Efferent CD8^+^ Tregs in ACAID act in the periphery, including in the eye, whereas afferent CD4^+^ Tregs act in the secondary lymphoid organs [11, 20]. In ACAID, eye-derived antigen presenting cells (APCs) induce the expansion of tolerogenic B cells in order to induce antigen-specific Tregs [21] and invariant natural killer T cells, which are additionally required for the generation of ACAID [22]. Furthermore, Hare et al. have also demonstrated that the anterior chamber injection of bovine interphotoreceptor retinoid-binding protein (IRBP) impaired the development of IRBP-specific delayed hypersensitivity and prevented the expression of experimental autoimmune uveoretinitis (EAU). This model of human uveitis can be induced by immunization of susceptible animals with a retinal antigen such as IRBP [23–25]. Moreover, the adoptive transfer of spleen cells obtained from mice that received IRBP to the anterior chamber suppressed and eliminated already established intraocular inflammation, which suggests that IRBP-specific, ACAID-inducing Tregs act on the efferent limb of the immune response [23]. A recent study has also shown that retinal antigen-pulsed tolerogenic APCs (ACAID-genic APCs) suppressed ongoing EAU by inducing CD8^+^ Tregs that, in turn, suppressed the effector activity of IRBP-specific T cells [26]. Thus, ACAID via antigen-specific Tregs suppresses IRBP-induced autoimmune uveoretinitis.

## 3. Generation of Tregs by an Immunosuppressive Intraocular Microenvironment That Includes Corneal Endothelium, Aqueous Humor, and Pigment Epithelial Cells

There is growing evidence that ocular resident cells, which include CE cells and PE cells, can contribute to the development and maintenance of the immunosuppressive intraocular microenvironment via the generation of Tregs [8]. In addition to the ocular PE cells, the eye also contains resident myeloid cell populations such as macrophages and microglial cells. However, most of the macrophages are restricted to the cornea and uveal tract, where they are responsible for maintaining homeostasis by removing debris and dead cells. Microglial cells also play important roles in retinal development/homeostasis and can mediate local neuroinflammatory reactions [27, 28].

Tregs induced by ocular PE cells, which constitutively express the transcription factor Foxp3, are indispensable for immune tolerance and homeostasis, as they suppress excessive immune responses that are harmful to the host [29]. Since Tregs have been involved in a series of pathologic processes associated with autoimmune disease and cancer [30, 31], Foxp3^+^ Tregs as well as Tregs in ACAID have been considered to be the key regulators in ocular immune privilege. In the following section, we describe the molecular mechanisms that underlie the Treg induction by ocular resident cells, in addition to evaluating the therapeutic potential of CE and PE-induced Tregs in helping to maintain the ocular immune privilege.

### 3.1. Strategy for Generation of Tregs by Ocular Resident Cells

We performed *in vitro* experiments to investigate whether cultured ocular resident cells, including CE, iris PE, ciliary body PE, and retinal PE (RPE) cells, would have the capacity to convert activated T cells into Tregs [8]. To generate Tregs *in vitro*, naïve CD4^+^ or CD8^+^ T cells obtained from C57BL/6 mice were cocultured with ocular PE cells in the presence of anti-CD3 antibody. T cells exposed to CE or PE cells were harvested, x-irradiated, and used as regulators (PE-induced Tregs). CD4^+^ T cells obtained from C57BL/6 mice were used as responder T cells. The responder T cells and PE-induced Tregs were then cocultured in the presence of anti-CD3 antibody in order to evaluate whether PE-induced Tregs suppressed the proliferation and cytokine production of the responder T cells. If there was suppression of the responder T cell activation, this would confirm that there was induction of Tregs by the ocular resident cells. The molecular mechanism underlying the generation of Tregs differs in accordance with the microenvironment of the ocular resident cells.

### 3.2. CE Cell-Induced Tregs

CE cells are part of the inner surface of the anterior chamber of the eye and come in contact with the aqueous humor. Human CE cells contribute to local immune tolerance in the human eye, as activated T cells exposed to CE cells fail to acquire effector T-cell function [32–34]. In addition, it has been reported that murine CE cells constitutively express various immunomodulatory molecules such as the Fas ligand, programmed death-ligand 1 (PD-L1/CD274), and glucocorticoid-induced tumor necrosis factor receptor family-related protein ligand, which leads to apoptosis of the effector T cells [35–37]. We have previously demonstrated that cultured human CE cells suppressed the activation of CD4^+^ Th1 cells in a cell contact-dependent manner via an interaction between the PD-1 and PD-L1 costimulatory molecules *in vitro* [34]. Subsequently, we then investigated whether human CE cells were capable of inhibiting T cells and generating Tregs *in vitro*. Cultured human CE cells produced enhanced membrane-bound active transforming growth factor beta 2 (TGF-*β*2) and suppressed activation of CD8^+^ T cells via a membrane-bound form of TGF-*β* [38]. Furthermore, cultured CE cells converted CD8^+^ T cells into Tregs via their membrane-bound active TGF-*β*. In addition, CE cell-induced CD8^+^ Tregs expressed both CD25^high^ and Foxp3 and suppressed activation of bystander effector T cells [38].

In a further experiment, we also examined whether murine CE cells have the capacity to generate Tregs. CD4^+^ T cells exposed to cultured murine CE cells expressed both CD25^high^ and Foxp3, with these T cells suppressing the activation of the bystander target T cells, which indicates that cultured murine CE cells have the capacity to generate Tregs [39]. Moreover, cytotoxic T lymphocyte-associated antigen-2 alpha (CTLA-2*α*: cathepsin L inhibitor), which is expressed on murine CE cells, promoted Tregs through TGF-*β* signaling [39]. Taken together, these findings suggest that cultured CE cells expressing TGF-*β* and CTLA-2*α* promote the generation of CD4/CD8^+^ Tregs that are able to suppress bystander effector T cells, thereby helping to maintain the immunosuppressive intraocular microenvironment.

### 3.3. Aqueous Humor-Induced Tregs

The aqueous humor participates in the local defense system of the eye and protects the intraocular tissue from immunogenic inflammation [6]. The aqueous humor contains immunosuppressive factors such as *α-*melanocyte-stimulating hormone (*α*-MSH), vasoactive intestinal peptide, and TGF-*β*2 [6]. It has been reported that the aqueous humor is capable of inducing Tregs via *α*-MSH and TGF-*β*2 [40, 41]. Furthermore, it has been reported that the aqueous humor obtained from rats recovering from monophasic EAU was able to enhance the regulatory function of ocular Tregs in recurrent EAU [42]. A recent study additionally showed that the aqueous humor promoted the conversion of naïve T cells into Foxp3^+^ Tregs, while TGF-*β* and retinoic acid had a synergistic effect on the Treg conversion mediated by the aqueous humor [43].

### 3.4. Ocular PE Cell-Induced Tregs

Ocular PE cells of the iris, ciliary body, and retina have been identified as important participants in creating and maintaining ocular immune privilege [8, 10, 44]. Iris PE cells have the capacity to suppress anti-CD3-driven activation of primed or naïve T cells [44]. We have previously shown that cultured iris PE cells suppressed TCR-driven T-cell activation *in vitro* through direct cell contact in which the B7-2 (CD86) expressed by the iris PE cells interacted with CTLA-4 on the responding T cells [45]. B7-2^+^ iris PE cells in the presence of anti-CD3 agonistic antibody supported selective activation of CTLA-4^+^CD8^+^ T cells that express their own B7-2 and secreted enhanced amounts of active TGF-*β*, leading to the global suppression of entire T-cell populations, including CD4^+^ T cells [46].

**Figure 1:**
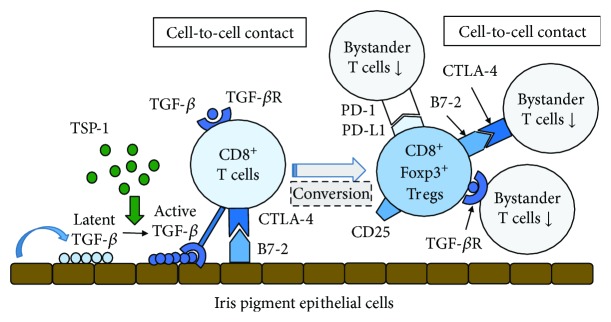
Molecular mechanism underlying the generation of regulatory T cells (Tregs) by murine iris pigment epithelial (PE) cells. Cultured iris PE cells suppress anti-CD3-driven T cell activation *in vitro* by direct cell contact in which B7-2 (CD86) expressed by iris PE cells interacts with cytotoxic T-lymphocyte antigen-4 (CTLA-4) on responding T cells. Furthermore, cultured iris PE cells expressing B7-2 induce the activation of CTLA-4^+^CD8^+^ T cells that express their own B7-2 and secrete enhanced amounts of active transforming growth factor beta (TGF-*β*), leading to the global suppression of entire T-cell populations including CD4^+^ T cells. Both iris PE cells and T cells exposed to iris PE cells upregulate their TGF-*β* and TGF-*β* receptor (TGF-*β*R) genes and suppress bystander T cells using membrane-bound or soluble TGF-*β*. In addition, iris PE cell-induced Foxp3^+^CD8^+^CD25^+^ Tregs suppress bystander T cells through cell contact via B7-2/CTLA-4 and/or programmed cell death- (PD-) 1/PD-L1 interactions. Thrombospondin-1 (TSP-1) produced from iris PE cells greatly contributes to the conversion of TGF-*β* from latent form to active form.

Subsequently, we then examined whether TGF-*β* was necessary for this process. Our study showed that both the iris PE and T cells exposed to iris PE cells were able to: (1) upregulate their TGF-*β* and TGF-*β* receptor genes, (2) convert the latent TGF-*β* they produced into the active form, and (3) use membrane-bound or soluble TGF-*β* to suppress bystander T cells. This demonstrated that both iris PE cells and B7-2^+^CTLA-4^+^CD8^+^ iris PE-induced Tregs produce enhanced amounts of active TGF-*β*, with the membrane-bound form of TGF-*β* used to suppress T-cell activation [47]. Furthermore, iris PE cells promoted the generation of Foxp3^+^CD8^+^CD25^+^ Tregs with cell contact via the B7-2/CTLA-4 interactions [48, 49]. In addition, iris PE-induced CD8^+^ Tregs greatly expressed PD-L1 costimulatory molecules and suppressed the activation of bystander Th1 cells that express PD-1 costimulatory receptor via a contact-dependent mechanism [50]. A previous study clearly demonstrated that thrombospondin-1 (TSP-1) binds and activates TGF-*β* [51]. Furthermore, iris PE cells generated CD8^+^ Tregs via TSP-1 and iris PE-induced CD8^+^ Tregs suppressed activation of bystander T cells via TSP-1 [52]. Taken together, these results strongly suggest that iris PE cell-induced CD8^+^ Tregs play a role in maintaining immune privilege in the anterior segment of the eye (Figure 1).

Previous studies have shown that the subretinal space is also an immune privileged site and that RPE cells act as immune privilege tissue [53, 54]. Moreover, RPE cells play pivotal roles in helping to maintain immune privilege in the subretinal space [3]. RPE cells have been shown to secrete soluble factors including TGF-*β*, TSP-1, and PGE_2_, which are mediators that alter the innate and adaptive immune responses [55–57]. Depending upon the inflammatory conditions, RPE cells are able to inhibit activated T cells that are regulated by the levels of the MHC class II expression [58]. Moreover, under the presence of inflammatory cytokines such as IL-17 and IFN-γ, RPE cells also highly express PD-L1, which can lead to suppression of the pathogenic activity of IRBP-specific T cells that induce EAU [59].

**Figure 2:**
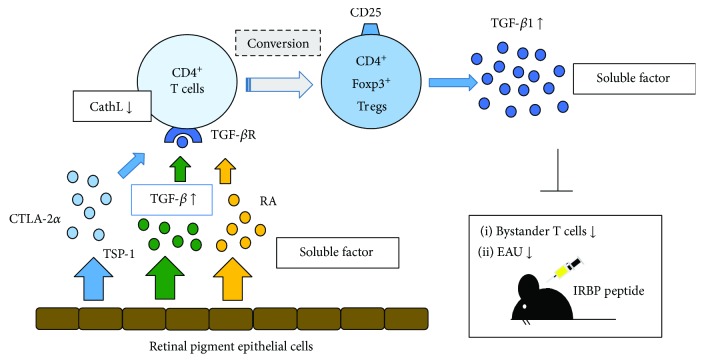
Molecular mechanism underlying the generation of regulatory T cells (Tregs) by murine retinal pigment epithelial (RPE) cells. RPE cells constitutively express cytotoxic T lymphocyte-associated antigen 2 alpha (CTLA-2*α*), a cathepsin L (CathL) inhibitor, which promotes the induction of Tregs. In addition, CD4^+^ T cells exposed to RPE cells predominantly express CD25 and Foxp3. CTLA-2*α*, thrombospondin-1 (TSP-1), and retinoic acid promote the development of CD4^+^CD25^+^ Foxp3^+^ Tregs by transforming growth factor beta (TGF-*β*) signaling *in vitro*. These Tregs produce high levels of TGF-*β* and suppress bystander T cells and experimental autoimmune uveoretinitis (EAU) induced by retinal antigen interphotoreceptor retinoid-binding protein (IRBP).

We have also reported that unlike for the iris PE cells, the RPE and ciliary body PE cells can suppress bystander T cells through inhibitory soluble factors and that the soluble form of the active TGF-*β*1/2 produced by the RPE and ciliary body PE cells demonstrated an immunosuppressive effect on the bystander T cells [56]. Subsequently, we then investigated whether RPE cell-exposed T cells could become Tregs *in vitro* and if the soluble form of TGF-*β* produced by the cultured RPE cells could convert T cells into Tregs. Our results showed that cultured RPE cells converted CD4^+^ T cells into Tregs in the presence of CTLA-2*α* [60]. RPE cells constitutively expressed CTLA-2*α* (cathepsin L inhibitor), which promoted the induction of Tregs, and CD4^+^ T cells exposed to RPE cells predominantly expressed CD25^+^ and Foxp3 [60]. Furthermore, recombinant CTLA-2*α* promoted the development of CD4^+^, CD25^+^Foxp3^+^ Tregs through TGF-*β* signaling *in vitro*, with these Tregs producing high levels of TGF-*β* [60]. These findings demonstrated that RPE cell-induced Tregs participated in the establishment of immune tolerance in the posterior segment of the eye (Figure 2). Our recent study also showed that RPE cells that produced retinoic acid and cultured RPE cells from vitamin A-deficient mice were unable to induce Foxp3^+^ Tregs [61]. These data are compatible with previous studies that have shown that the conversion of naïve T cells into Foxp3^+^ Tregs in the eye required TGF-*β* and retinoic acid [43, 61]. Thus, overall, these findings indicate that TGF-*β* and retinoic acid interact to induce Tregs for immunological regulation in the eye (Figure 2).

## 4. Immunomodulation of Uveitis by Tregs

Thymus-derived naturally occurring Tregs play an essential role in preventing autoimmune disease, with depletion of the naturally occurring Tregs leading to multiorgan autoimmune disease [29, 30]. Indeed, depletion of CD4^+^CD25^+^ T cells before immunization has been shown to exacerbate the murine EAU model of human uveitis [62]. A recent study reported that retinal antigen-specific Foxp3^+^ Tregs play a role in the natural resolution of EAU and the maintenance of remission [63]. Conversely, there is growing evidence that administration of Tregs can effectively suppress uveitis in mice. Antigen-specific Tregs generated by *α*-MSH and TGF-*β*2 have also been shown to suppress EAU [64]. In addition, lipopolysaccharide-activated dendritic cell-induced CD4^+^CD25^+^Foxp3^+^ Tregs inhibit CD4^+^CD25^−^ effector T cells, and when adoptively transferred, these Tregs suppress EAU [65]. Moreover, intravenous administration of antigen-specific Tregs has the capacity to control uveitis in mice [66]. In addition, an intravitreous injection of preactivated polyclonal Tregs was also shown to suppress uveitis in mice [67]. In our own study, we also demonstrated that the adoptive transfer of CD4^+^CD25^+^ natural Tregs ameliorated the development of EAU [68]. However, the ability to prepare large numbers of Tregs for adoptive transfer and stable expression of Foxp3 *in vivo* remains problematic.

Since retinoic acid has been reported to contribute to high and stable Foxp3 expression via the retinoic acid receptor in the presence of TGF-*β* [69], we investigated whether retinoic acid has the capacity to expand Tregs and ameliorate the development of EAU. The results of our study demonstrated that retinoic acid promoted the generation of CD4^+^Foxp3^+^ Tregs in the presence of TGF-*β*, with systemic administration of retinoic acid during the induction phase reducing the clinical score of EAU [70, 71]. Furthermore, oral administration of a novel synthetic retinoic acid, Am80, not only increased the frequency of Tregs in draining lymph nodes in mice with EAU but also suppressed the Th1/Th17 response [71]. Am80 is more stable to light, heat, and oxidation than retinoic acid, and Am80 is clinically available in Japan for the treatment of relapsed acute promyelocytic leukemia. Thus, systemic administration of retinoid may not only have the potential to promote the expansion of Tregs *in vivo*, but it appears that it may also have therapeutic possibilities. In addition, since a previous report demonstrated that TGF-*β* levels were significantly elevated in the aqueous humor from EAU eyes [72], it is conceivable that the expression of Foxp3 on intraocular T cells in Am80-treated mice may be increased, with expansion of Foxp3^+^ Tregs possibly contributing to the amelioration of murine EAU.

Stabilization of Foxp3 expression is necessary for the generation and maintenance of highly suppressive Tregs *in vivo* for clinical use. Presently, various reagents and drugs, such as rapamycin, IL-2, and retinoic acid, have been reported to stabilize Foxp3 expression [73]. Furthermore, epigenetic modification of Foxp3 expression may be required in order to generate stable Tregs for clinical application [74].

As described above, we demonstrated that recombinant CTLA-2*α* (rCTLA-2*α*) derived from RPE cells has the capacity to generate Tregs through the promotion of TGF-*β* production [60]. Indeed, rCTLA-2*α*-treated mice had a high population of Foxp3^+^ Tregs compared with CD4^+^ T cells from control EAU mice [75]. Furthermore, the severity of EAU was significantly reduced in rCTLA-2*α*-treated mice and cathepsin L-deficient mice as compared with wild type mice. Thus, these findings suggest that CTLA-2*α* secreted from RPE cells converts intraocular effector T cells into Foxp3^+^ T cells that then acquire regulatory functions and lead to the amelioration of ocular inflammation [75].

We next assessed the ability of murine RPE cell-induced Tregs to suppress EAU in mice through the use of adoptive transfer. Our data revealed that the administration of RPE cell-induced Tregs that greatly expressed Foxp3 were able to suppress ocular inflammation in mice with EAU [76]. Moreover, the retinal antigen-specific cytokine response (IFN-*γ* and IL-17) was reduced when intraocular T cells were cocultured with RPE cell-induced Tregs *in vitro* [76]. These findings suggest that RPE cell-induced Tregs might possibly have a therapeutic potential for the treatment of autoimmune uveoretinitis.

Another recent challenge encountered with Treg therapy was reported while using a murine ocular inflammatory model, which included both antigen-specific and nonantigen-specific murine disease models [67]. In the antigen-specific model, TCR-hemagglutinin (HA) transgenic mice and HA-specific effector T cells were used to induce uveitis in mice in which HA is constitutively expressed in the retina. The authors found that Treg transplantation in the systemic circulation significantly suppressed local ocular inflammation. Moreover, polyclonal Tregs that expanded ex vivo also significantly improved ocular inflammation when these Tregs were injected locally, that is, intravitreally. Other recent investigations have additionally shown that several regulatory molecules including IL-22, aryl hydrocarbon receptor, and CD73/adenosine contribute to the generation of Tregs/regulatory mesenchymal stem cells to control EAU in mice [77–79]. These murine study findings support the concept of Treg therapy for ocular inflammation and are the foundation for further human clinical trials.

## 5. Ocular Surface Disease and Tregs

Dry eye disease (DED) is one of the major ocular surface inflammatory disorders [80, 81]. It is well known that activation and infiltration of pathogenic immune cells, primarily CD4^+^ T cells, contribute to the development of ocular surface inflammation in DED [82–84]. Increased IL-17 and IFN-*γ* have been observed in both clinical and experimental DED [85–89]. Recent studies have demonstrated that Th17 cells are the principal effectors actively mediating DED [90, 91]. In fact, Chauhan et al. reported that while Treg frequencies remained unchanged, there was a marked decrease in their potential to suppress the effector Th17 cells in a mouse model of DED. This suggests that dysfunction of Tregs can be presumed to be one of the major causes in ocular anterior segment inflammation such as DED [90, 92]. It has also been reported that *in vitro-*expanded Foxp3^+^ Tregs maintain a normal phenotype and are capable of suppressing immune-mediated ocular surface inflammation in animal models, with *in vitro*-expanded Tregs able to more efficiently reduce tear cytokine levels and conjunctival cellular infiltration compared to freshly isolated Tregs [93]. Antigen specificity is one of important factors required for Tregs in order to more effectively regulate the pathogenic inflammatory cells. Presently, while the specific autoantigen responsible for the induction of dry eye disease has yet to be identified, it has been suggested that *α*-fodrin might be a candidate autoantigen in primary Sjögren's syndrome [94]. Identification of a specific autoantigen in DED could potentially lead to the generation of antigen-specific Tregs that ultimately could become a promising therapy for immune-mediated ocular surface inflammation.

## 6. Corneal Transplantation and Tregs

The three fundamental factors that contribute to corneal allograft survival are (1) blocking the induction of the immune response against allograft antigens, (2) generation of Tregs that can suppress the destructive alloimmune reaction, and (3) induction of apoptosis of inflammatory cells at the graft/host interface [95]. Long-term corneal allograft survival leads to an antigen-specific suppression of the delayed type hypersensitivity immune response and resembles the suppression of the delayed type hypersensitivity that is observed in ACAID [96]. Cunnusamy et al. have reported that there are two different Tregs that can promote corneal allograft survival. These include (1) CD4^+^CD25^+^ Tregs induced by the corneal allograft act at the efferent arm of the immune response in order to suppress the delayed type hypersensitivity and (2) CD8^+^ Tregs induced by anterior chamber injection of alloantigens to suppress the efferent phase of the immune response [95]. Furthermore, it has also been demonstrated that the levels of Foxp3 expression in Tregs from corneal allograft acceptors were significantly higher compared to that seen in Tregs from the corneal allograft rejectors, which suggests that dysfunction of Tregs can be presumed to be one of the major causes of corneal allograft rejection [97]. Moreover, Tregs of allograft acceptors during adoptive transfers were reported to significantly increase the allograft survival rate [97]. In addition, it was also shown that the presence of allospecific Tregs in graft recipients primarily suppressed the induction of alloimmunity in the regional draining lymph nodes rather than suppressing the effector phase of the immune response in the periphery [97]. Hori et al. have shown that the expression of the glucocorticoid-induced tumor necrosis factor receptor family-related protein ligand (GITRL) in the cornea led to the local expansion of Foxp3^+^CD4^+^CD25^+^ Tregs, thereby contributing to the immune privilege status for the corneal allografts [98]. As previously described, we have demonstrated that cultured CE cells expressing TGF-*β* and CTLA-2*α* promote the generation of CD4/CD8^+^ Tregs that are able to suppress the bystander effector T cells [39]. Taken together, these findings suggest that cell therapy performed when using Tregs may potentially be able to promote corneal allograft survival during transplantation. However, other recent evidence has shown that there is an increased risk of corneal allograft rejection in mice with allergic conjunctivitis and impaired function of the peripherally induced regulatory T cells in hosts who were at a high risk of graft rejection [99–101]. In fact, increases in corneal graft rejection were found in hosts reported to have previous ocular allergies during routine clinical practice examinations due to allergic inflammatory responses [102, 103]. A recent study demonstrated that systemic treatment of high-risk recipient mice with low-dose IL-2 led to an expansion and improved suppressive function of Tregs, reduced leukocyte infiltration of the graft, and promotion of corneal allograft survival [104]. Further studies that help to better clarify the mechanism of the generation and function of Tregs in corneal allograft transplantation will hopefully lead to the promotion of ocular immune privilege and survival of the corneal allograft in hosts with inflamed or vascularized recipient beds after Treg-based therapy.

## 7. Current Concept and Strategy of Treg Therapy in Humans

Adoptive transfer of Tregs in humans has been examined and tested in order to treat systemic autoimmune diseases or posttransplant-related complications [105, 106]. These pathologic states are partly caused by the dysfunction of Tregs or due to the relative inferior activity of Tregs to effector T cells. Restoration or reinforcing immune regulation by Tregs is the primary aim of the treatment. Furthermore, there is clear evidence for a relationship between the dysfunction of Tregs and autoimmune disease onset. The Foxp3 gene is mutated in immune dysregulation, polyendocrinopathy, enteropathy, and X-linked syndrome. Thus, Foxp3^+^ Tregs are thoroughly absent throughout the whole body, which can cause fatal autoimmunity leading to death during the early stages of life if hematopoietic stem cell transplantation is not performed [107, 108]. These examples demonstrate that Treg deficiency or relative dominant proinflammatory cytokines are in fact related to the autoimmune disease onset. Consequently, we believe that adoptive transfer of Tregs into affected patients will be a promising strategy against this pathological inflammation.

In order to achieve new therapeutic strategies for clinical application, the critical issues that need to be addressed include the following: (1) human Treg phenotypes need to be characterized in detail in order for clinical application, (2) techniques need to be standardized for isolating and expanding Tregs in order to avoid contamination, and (3) a method for delivering Tregs into patients will need to be established. With regard to the first issue, appropriate characteristics of Tregs will not be identical for each disease. Presently, the use of antigen-specific Tregs is an ideal choice for cases in which the target antigen is already known. However, the causative self-antigen remains unknown in most autoimmune diseases. Furthermore, it is practically impossible to cover all antigen repertoires in autoimmune diseases. Therefore, a more realistic idea for addressing this issue would be to utilize polyclonal non-antigen-specific Tregs, which may suppress inflammation in a bystander manner. Although polyclonal Tregs may have a relatively broad suppressive function, the effectiveness of polyclonal Tregs is still unclear and could potentially differ for individual organs and disorders.

For the second issue, there are several potential sources of Tregs. Autologous peripheral blood is a straightforward choice, as it is easy to collect. In addition, allogeneic umbilical cord blood, preferably HLA matched, is a favorable alternative choice [109]. Moreover, in the case of graft-versus-host disease (GVHD), allogeneic donor-derived Tregs are also usable material. Regardless of the Treg source, the next critical technical step is the sorting of the polyclonal Tregs according to the characteristic surface markers. CD4^+^CD25^+^ selection is the most common method. Although Foxp3 is the most Treg-specific marker, it can only be detected by permeabilization, which, unfortunately, causes cell death. Therefore, this procedure cannot be used for the purpose of selection. In addition to CD4^+^CD25^+^ selection, cells are commonly sorted according to CD127^low^ expression for further purification [110].

Human Foxp3^+^ Tregs have recently been categorized based on CD25 and CD45RA expression, with CD25^low^CD45RA^+^ expression indicating resting Tregs, CD25^low^CD45RA^−^ expression indicating nonsuppressive Tregs, and CD25^high^CD45RA^−^ expression indicating active Tregs. However, for these treatments, it has been suggested that CD25^high^CD45RA^−^ Tregs might be the best population to use [111, 112]. After sorting the specific populations, cell expansion is essential because the numbers of circulatory Tregs are relatively small (up to 5–7% of CD4^+^ T cells) [110].

Costimulation with anti-CD3/CD28 and IL-2 stimulation is a popular technique among the general expansion protocols [106]. This protocol enables expansion by a few hundredfold at most. However, contamination with T cells other than Tregs is unpreventable to some extent following this massive expansion. While the acceptable amount of contamination for clinical use remains uncertain, it may be dependent on the target disease. From this point of view, the use of umbilical cord blood-derived T cells, which constitute naïve cells, may be advantageous since natural Tregs are used [113], thereby avoiding contamination of the memory effector T cells in the injected cells.

For the third issue, there are many options for delivering expanded cells. Systemic injection via peripheral circulation is common, while local administration is also possible in some organs. However, careful attention should be paid to potential infusion reactions that could occur following administration via blood circulation. Even so, the eye is one of the best target organs for local administration. Inflammatory disease in the eye, such as uveitis, is the next challenge for targeted Treg therapy [76].

### 7.1. Application of Tregs in Treatment of Ocular Inflammation

Since the cause of noninfectious uveitis is diverse, most disorders can be treated or well controlled with immunosuppression. As a result, noninfectious uveitis can be viewed as an autoimmune disease of the eye. Systemic or topical administration of steroids has long been used as major immunosuppressive therapies for ocular inflammation. In addition to steroids, immunosuppressive agents or recently introduced monoclonal antibodies against inflammatory cytokines are also frequently administered in these patients [114]. In uveitis, proinflammatory cytokines from pathological T cells play central roles in the inflammation [115, 116]. We previously reported the decreased frequency of peripheral Tregs in patients with active uveitis such as Behçet's disease [117]. In healthy individuals, organ homeostasis is maintained by central and local tolerance [118]. As previously mentioned, the eye is one of the major immune privileged sites [10], where ocular PE cells play a central role in developing local tolerance [9]. The breakdown of immune tolerance leads to unfavorable autologous antigen-specific attacks against organs by the effector T cells. Failure in immune tolerance is partly due to Treg dysfunction and/or dominant effector T cell activity. Since noninfectious uveitis is considered an autoimmune disease, it is logical to assume that adoptive transplantation of Tregs should inhibit ocular inflammation. Thus, restoration of Treg function or artificial transfer of Tregs into noninfectious uveitis patients is likely to be a promising therapeutic choice for treating this disease.

Based on the therapeutic effect of Tregs in animal models of autoimmune uveitis [67], a phase I/II clinical trial has been started in Europe in patients with severe bilateral uveitis who are refractory to standard treatments and presented with a low visual acuity [119, 120]. The objective of this trial is to evaluate the safety of an intravitreal injection of ex vivo-activated polyclonal Tregs in patients with refractory and end-stage noninfectious uveitis. The result of this ongoing clinical trial and further studies on the safety and efficacy of Tregs should provide valuable information for the application of Tregs in patients with refractory uveitis.

### 7.2. Establishment of Tregs by Ocular Microenvironment

Similar to our previous murine studies, human ocular PE cells have been shown to have immunosuppressive functions, which form the immune privilege in the eye [121]. Primary cultured human iris PE cells are able to suppress the activation of bystander responder T cells *in vitro* [122]. Human iris PE cells suppress cell proliferation and cytokine production by responder T cells via direct cell-to-cell contact in a TGF-*β*-dependent manner. Furthermore, responder T cells are not only conventional autogenic activated T cells but also allogeneic activated T cells or T cell clones that have been established from uveitis patients [122]. In addition, human CE cells have an immunoregulatory function equivalent to that of human iris PE cells [38]. Interestingly, human CE cells can inhibit activated PD-1^+^ helper T cells via the PD-L1–PD-1 interaction [34], while activated T cells are suppressed via membrane-bound TGF-*β* [38]. Thus, human iris PE cells and CE cells cooperatively create immune privilege in the anterior chamber.

Focusing on the posterior ocular segment, human RPE cells show potent regulatory function in ocular inflammation as well. Inflammatory cells in the retina, where cells cannot freely move, do not always come in direct contact with RPE cells. However, RPE cells can regulate inflammation by secreting soluble inhibitory molecules or generating Tregs [9]. Similar to the results of previous murine studies, human RPE cells have shown a great ability to generate Tregs *in vitro* [121]. RPE-induced Tregs strongly suppress cytokine production and proliferation of intraocular T-cell clones derived from active uveitis patients. CD4^+^ T cells express CD25 and Foxp3 after culture with RPE supernatants, especially TGF-*β*2-pretreated RPE cells. The suppressive mechanism of human RPE-induced Tregs is mediated in a TGF-*β*-dependent manner, similar to that observed for murine RPE-induced Tregs. Based on these results, practical application of RPE-induced Tregs for treating uveitis and transplantation of a retina/RPE graft in patients with retinal degeneration appears to be a logical approach. However, for future clinical applications of Tregs in inflammatory ocular diseases and retina/RPE transplantation, there needs to be further optimization of the establishing and expanding of Tregs.

**Figure 3:**
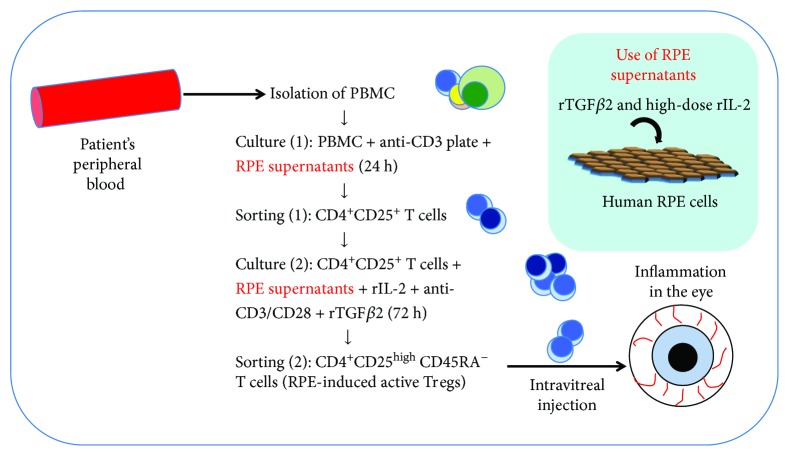
Regulatory T-cell (Treg) therapy in ocular disease: the original source of Tregs is the patient's peripheral blood. Following isolation of peripheral blood mononuclear cells (PBMCs) from the blood, PBMCs are cultured on anti-CD3-coated plates with RPE supernatants for 24 h. RPE supernatants are collected from culture media of human RPE cell lines with transforming growth factor beta 2 (TGF-*β*2) and high-dose interleukin 2 (IL-2). CD4^+^CD25^+^ T cells are first selected from cultured PBMCs. Sorted CD4^+^CD25^+^ T cells are then recultured with RPE supernatants together with recombinant IL-2 (rIL-2) and anti-CD3/CD28 antibodies for 72 h. In the final sort, CD4^+^CD25^high^CD45RA^−^ T cells are collected, which are best suited for intravitreal injection into uveitis patients.

Based on these previous studies, we subsequently developed a method that could be used to more selectively and efficiently obtain RPE-induced Tregs (Figure 3). With this method, PBMCs are first cultured with recombinant TGF-*β*2-pretreated RPE supernatant on an anti-CD3-coated plate. CD4^+^CD25^+^ T cells are then sorted and recultured together with high-dose recombinant IL-2, antihuman CD3/CD28 antibodies, and TGF-*β*2 for 3 days. Using this method, it is possible to produce a large amount of CD25^high^CD45RA^−^ active Tregs that highly express Foxp3, CTLA-4 (CD152), and tumor necrosis factor receptor superfamily 18 (TNFRSF18). Furthermore, these RPE-induced Tregs secrete large amounts of suppressive cytokines TGF-*β*1 and IL-10 and suppress bystander target Th1 cells or Th17 cells [76].

## 8. Conclusions and Future Directions

Although CE and RPE cells are responsible for maintaining the homeostasis of the microenvironment of the eye, they also have unique anti-inflammatory and immunogenic roles in inflammation. Both ocular resident mesenchymal cells and peripheral tolerance of ACAID actively contribute to the regulation of immune responses via the generation of Tregs. These eye-specific Tregs have the therapeutic potential for not only autoimmune uveoretinitis but also promoting allograft survival after transplantation. At present, the therapeutic potential of Tregs in humans has been both examined and tested in order to treat systemic autoimmune diseases or posttransplant-related complications. However, further studies will be required in order to establish Treg therapy for active noninfectious uveitis. In addition, a better understanding of the molecular mechanism that regulates ocular immune privilege may lead to an effective therapeutic strategy that can be used to target individual patients with refractory uveitis.
